# Evaluation of the Distribution and Elimination of Balanced Isotonic Crystalloid, 5% Hypertonic Saline, and 6% Tetrastarch 130/0.4 Using Volume Kinetic Modeling and Analysis in Healthy Conscious Cats

**DOI:** 10.3389/fvets.2020.587564

**Published:** 2020-11-13

**Authors:** Xiu Ting Yiew, Shane W. Bateman, Robert G. Hahn, Alexa M. E. Bersenas

**Affiliations:** ^1^Department of Clinical Studies, Ontario Veterinary College, University of Guelph, Guelph, ON, Canada; ^2^Research Unit, Södertälje Hospital, Södertälje, Sweden; ^3^Karolinska Institutet Danderyds Hospital (KIDS), Stockholm, Sweden

**Keywords:** distribution, elimination, fluid therapy, volume kinetics, crystalloid, hypertonic saline, tetrastarch, cats

## Abstract

This prospective, randomized, blinded, interventional cross-over study investigated the distribution, elimination, plasma volume expansion, half-life, comparative potency, and ideal fluid prescription of three commonly prescribed intravenous (IV) fluids in 10 healthy conscious cats using volume kinetic analysis that is novel to veterinary medicine. Each cat received 20 mL/kg of balanced isotonic crystalloid (PLA), 3.3 mL/kg of 5% hypertonic saline (HS), and 5 mL/kg of 6% tetrastarch 130/0.4 (HES) over 15 min on separate occasions. Hemoglobin concentration, red blood cell count, hematocrit, heart rate, and blood pressure were measured at baseline, 5, 10, 15, 20, 30, 40, 50, 60, and every 15 min until 180 min. Urine output was estimated every 30 min using point-of-care bladder ultrasonography. Plasma dilution derived from serial hemoglobin concentration and red blood cell count served as input variables for group and individual fluid volume kinetic analyses using a non-linear mixed effects model. In general, the distribution of all IV fluids was rapid, while elimination was slow. The half-lives of PLA, HS, and HES were 49, 319, and 104 min, respectively. The prescribed fluid doses for PLA, HS, and HES resulted in similar peak plasma volume expansion of 27–30%. The potency of HS was 6 times higher than PLA and 1.7 times greater than HES, while HES was 3.5 times more potent than PLA. Simulation of ideal fluid prescriptions to achieve and maintain 15 or 30% plasma volume expansion revealed the importance of a substantial reduction in infusion rates following initial IV fluid bolus. In conclusion, volume kinetic analysis is a feasible research tool that can provide data on IV fluid kinetics and body water physiology in cats. The rapid distribution but slow elimination of IV fluids in healthy conscious cats is consistent with anecdotal reports of fluid overload susceptibility in cats and warrants further investigation.

## Introduction

The clinical practice of intravenous (IV) fluid therapy in human and veterinary medicine is changing based on new findings (1–3) as well as ongoing debate (4) regarding ideal fluid choice, dose, rate, efficacy, and safety of fluid therapy (4–6). Limited veterinary scientific literature on IV fluid therapy (7) has given rise to empirical fluid therapy recommendations that are based on broad assumptions, historical physiological principles, clinician's anecdotal experiences, or extrapolation from human clinical trials and canine experimental models. Extrapolation of data across species is less than ideal as there may be critical species variations in body water physiology and fluid dynamics associated with different IV solutions. Fluid therapy has the potential to result in fluid overload that increases morbidity and mortality (8, 9), especially in cats (10), yet little evidence exists to support current fluid administration practices in this species (7). The landmark publication reporting increased mortality following standard fluid boluses in African children with severe infection (11) took the medical profession by surprise and subsequently raised many questions regarding our understanding of fluid pharmacokinetics (PK). Since then, there has been a momentous shift in fluid therapy paradigms toward fluid stewardship (12), context-sensitive fluid therapy (13), and treating fluids as drugs (3, 5). Recently, the diverse elimination half-lives (*t*_1/2_) of balanced isotonic crystalloid and colloid solutions, administered under a wide variety of physiological conditions, have provided further scientific evidence of the importance of context-sensitive fluid therapy (14).

For over two decades, a group of researchers has developed and refined an innovative PK model adapted for body fluid spaces known as volume kinetic (VK) modeling (15–20), providing a mathematical research platform to understand the effects of IV fluid administration on body fluid spaces. Volume kinetics, or in simplistic terms PK of IV fluids, is able to provide descriptive data on the distribution and elimination of IV fluids administered under various physiological conditions in expressions comparable to those employed in conventional PK (18). Visual inspection of the plasma dilution-time curves provides information on the magnitude and time course of plasma volume expansion during and following actual IV fluid infusion (16). Using generated VK parameter estimates, subsequent computer simulation of plasma dilution-time curves allows insight into how IV fluid therapy should be performed (17, 18, 20). With increasing emphasis on safe and responsible fluid prescription, VK is gaining recognition (12, 13) as IV fluids can now be studied like pharmaceutical drugs leading to improved understanding of their time-volume effects or *t*_1/2_ on plasma and interstitial fluid compartments (14), making evidence-based approaches to fluid therapy possible.

**Figure 1:**
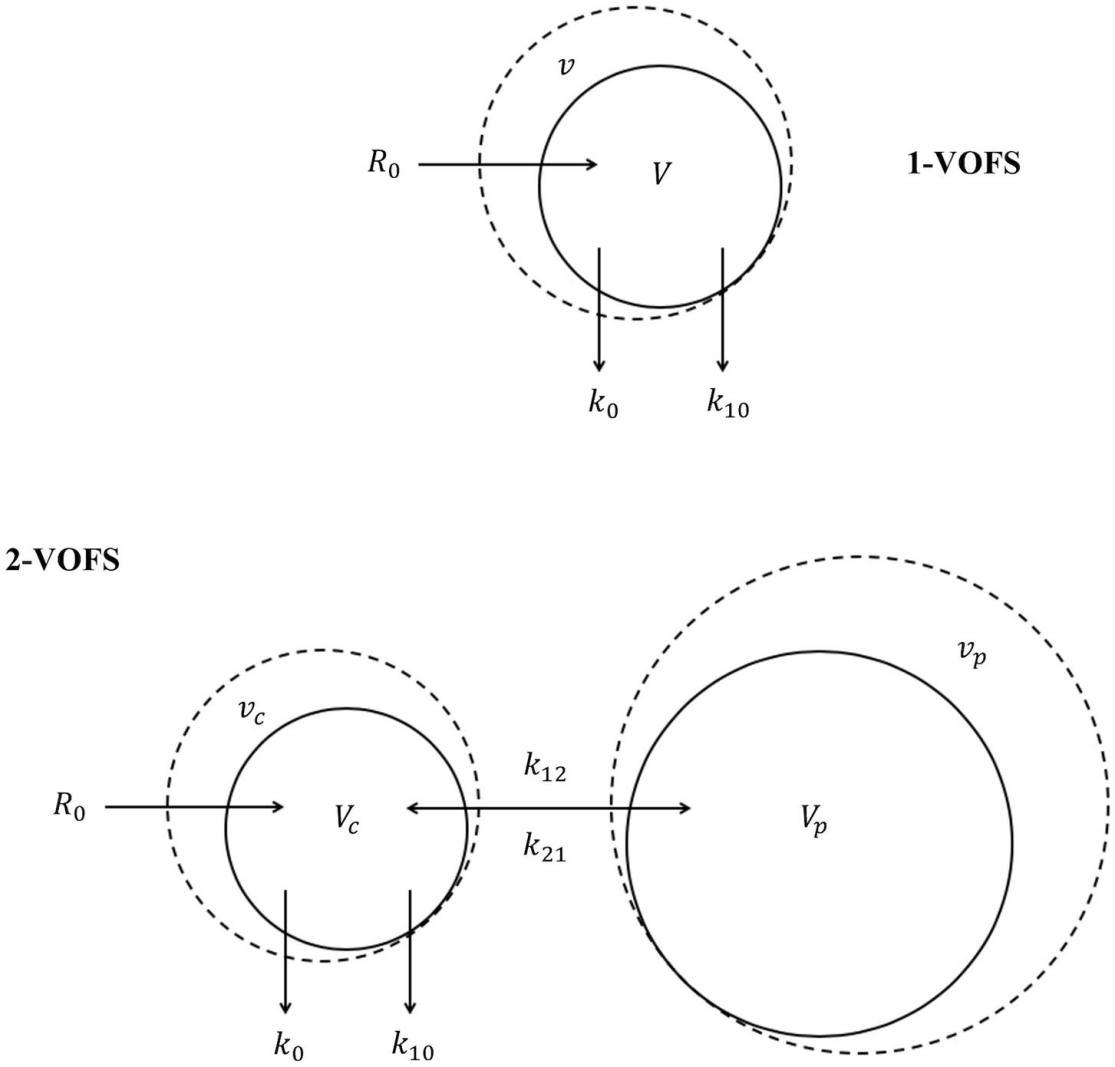
One-volume fluid space (1-VOFS) and two-volume fluid space (2-VOFS) micro-constant kinetic models. Adapted from previous VK work (21–23).

The principles of VK are beyond the scope of this manuscript and readers are directed to current reviews (14, 17, 18, 20). In essence, VK models are designed based on the notion that IV fluid infused at a prescribed rate (*R*_0_) expands the volume of expandable body fluid space (*V*). Intravenous fluid alters the volume of expanded body fluid space (*v*) over time as IV fluid is distributed and eliminated from the body fluid space. Elimination occurs in proportion to volume expansion (*v* − *V*) governed by a first-order elimination rate constant (*k*_10_). Distribution of IV fluids can be described using kinetic models analogous to the compartmental models in PK analysis. Intravenous fluid can distribute within a single body fluid space, or be distributed between two body fluid spaces, the central (*v*_*c*_) and peripheral (*v*_*p*_) compartment, in proportion to volume expansion of each respective body fluid space governed by first-order bi-directional intercompartmental rate constants (*k*_12_ and *k*_21_). The schematic diagrams of the one-volume fluid space (1-VOFS) and two-volume fluid space (2-VOFS) kinetic models are shown in Figure 1.

Although VK has been studied extensively in humans and animal research models (14, 17, 18, 20), this concept is novel in companion animal species and scientific literature is not available. Hence, this preliminary study was designed to answer the fundamental research question of whether VK modeling can be utilized in cats to study IV fluid kinetics and body water physiology in this species. The primary objective of this study was to investigate the distribution and elimination of three commonly prescribed IV fluids [i.e., balanced isotonic crystalloid (PLA), 5% hypertonic saline (HS), and 6% tetrastarch 130/0.4 (HES)] in healthy conscious cats using VK modeling and analysis. The secondary objective was to describe the plasma volume expansion, half-lives, potency, and ideal fluid prescriptions of PLA, HS, and HES. We hypothesized that VK modeling can be applied to healthy cats in a research setting through demonstration of similar peak plasma volume expansion effect when approximately equipotent doses of the aforementioned IV fluids were administered. We further hypothesized that the plasma dilution-time curve of PLA, HS, and HES would be different based on the conventional understanding of the physiological behavior of balanced isotonic crystalloids, hypertonic crystalloids, and synthetic colloids in the mammalian body.

## Materials and Methods

This prospective, randomized, blinded, three-treatment and three-period (3 × 3) cross-over experimental study was performed at the Ontario Veterinary College, University of Guelph. This study, which required the sampling of ~24 mL of blood per cat over a duration of 3 weeks, was approved by and conducted in accordance with the guidelines of the University of Guelph's Animal Care Committee. Ten male intact purpose-bred domestic shorthair cats were enrolled into this study between June and July 2017. Each cat was certified healthy based on physical examination, complete blood count, serum biochemistry profile, total thyroxine level, urinalysis, symmetric dimethylarginine level, and N-terminal pro B-type natriuretic peptide level. All cats underwent a 7-days acclimatization period and were conditioned to tolerate general handling, simple restraint techniques, and wearing an anti-anxiety shirt^1^ as well as light neck bandages. Throughout the entire study period, these cats were fed a commercial dry kibble and had *ad-libitum* access to fresh water.

### Instrumentation

Two days prior to the experiment, each cat was instrumented with a central venous jugular catheter^2^ under general anesthesia. Sedation included butorphanol^3^ (0.4 mg/kg IM) and dexmedetomidine^4^ (3 μg/kg IM), followed by propofol^5^ induction (2–3 mg/kg IV) and maintenance of general anesthesia using isoflurane^6^ in oxygen. Following placement, the central venous catheters were flushed and locked with unfractionated heparin^7^ (100 U/mL) according to catheter volume (0.23 mL). Sedation was reversed with atipamezole^8^ (equal volume to dexmedetomidine^4^ IM). The catheter sites were inspected daily, cleaned as needed with aqueous chlorhexidine gluconate 0.05% solution^9^, and the heparin^7^ lock was replaced each day throughout the study period. When difficulty or inability to aspirate the catheter was encountered, forceful flushing of the catheter with small volumes of 0.9% normal saline was first attempted. If saline flush was unsuccessful in restoring catheter flow, tissue plasminogen activator^10^ (1 mg/mL) was instilled into the partially occluded catheter lumen (0.23 mL) and aspiration was reattempted after a 1-h dwell time. When required, replacement of central venous catheters was performed at least 24 h prior to the next scheduled experiment using similar technique and general anesthesia protocol.

### Experimental Procedure

Food and water were withheld during each 3-h experimental period. Each cat received 50 mg (≤5 kg body weight) or 100 mg (>5 kg body weight) trazodone^11^ orally followed by 0.4 mg/kg butorphanol^3^ intravenously 90 min later. Following light sedation, a 22-gauge, 1-inch IV catheter was established in the cephalic vein. A low stress environment was maintained throughout the entire experiment with cats wearing an anti-anxiety shirt^1^, using feline pheromone^12^, and implementation of light and noise reduction.

Each cat received three randomly assigned IV fluid boluses on three separate days with a minimum 72-h washout period between treatments. Intravenous fluid treatment consisted of 20 mL/kg of balanced isotonic crystalloid solution^13^ (PLA), 3.3 mL/kg of 5% sodium chloride solution^14^ (HS), and 5 mL/kg of 6% tetrastarch 130/0.4 solution^15^ (HES) administered over 15 min through the peripheral catheter using a calibrated fluid pump^16^. The fluid doses were selected on the basis of approximately similar volume expansion effect (16). The sodium contents of the prescribed PLA and HS doses were similar, while the prescribed HES dose was based on a 1:4 ratio to crystalloid infusion (24). Subject order and treatment sequences were randomized by an individual independent to the study using an online research randomizer^17^. Fluid delivery was provided by individuals independent to the study such that investigators remained blinded to fluid treatments until data analysis.

### Measurements and Data Collection

Duplicate blood samples (0.4 mL × 2) were collected at baseline prior to each fluid administration. Subsequently during IV fluid infusion, single blood samples were collected every 5 min for the first 20 min, then every 10 min until the 60-min time point, and every 15 min thereafter until completion of the 3-h observation period (total of 18 blood samples). At each blood sampling occasion, 0.4 mL of whole blood was collected into a 500 μL ethylenediaminetetraacetic acid (EDTA) microtainer tube and a total of 0.4 mL of saline flush was returned to the cat. Blood samples were collected from the central venous catheter using a three-syringe technique as follows: instillation of 0.2 mL of saline flush followed by aspiration of at least 0.5 mL of diluted blood sample, collection of desired blood sample, then return of initial diluted blood sample followed by an additional 0.2 mL of saline flush. Hemoglobin (Hb) concentration, red blood cell (RBC) count, and hematocrit (HCT) were analyzed by an automated hematology analyzer^18^ of an accredited laboratory within 4 h from each blood draw.

At similar time points for blood sampling, systolic arterial pressure (SAP), diastolic arterial pressure (DAP), mean arterial pressure (MAP), and heart rate (HR) were recorded using an oscillometric blood pressure device^19^. During the study, paired sets of longitudinal and transverse cysto-colic ultrasound images were acquired for each cat positioned in dorsal recumbency at baseline and every 30 min throughout the experiment. Urine output measurements were obtained using a novel non-invasive 3-dimensional bladder volume estimation method described elsewhere (25). The average bladder volume estimations from two investigators were used to estimate urine output.

### Plasma Dilution Determination

The average coefficients of variation (CV) for Hb concentration and RBC count were determined using the respective duplicate baseline measurements (16). The mean of the duplicate baseline measurements served as the initial baseline values for that experiment. The average plasma dilutions obtained from serial Hb concentrations and RBC counts were calculated using the equation (18, 26):

(1) $$\begin{array}{l} \text{ Plasma dilution},\;\frac{v_{\left(t\right)}-V}{V}\\ \begin{array}{l} =\left[\frac{Hb_{\left(0\right)}/Hb_{\left(t\right)}-1}{1-HCT}+\frac{RBC_{\left(0\right)}/RBC_{\left(t\right)}-1}{1-HCT}\right]÷2 \end{array} \end{array}$$

These calculations were performed using a computer software^20^. To determine whether Hb correction was necessary, three randomly selected experimental data sets were analyzed using plasma dilution calculated from corrected (18) and uncorrected Hb concentrations. The VK data output utilizing plasma dilution with and without Hb correction were compared, and the differences were found to be marginal. Therefore, the reported VK analyses were performed using the uncorrected plasma dilution.

### Volume Kinetic Modeling

All data sets, regardless of fluid type, were first analyzed together, and both 1-VOFS and 2-VOFS kinetic models were fitted to the data. Following that, data sets were analyzed separately according to the different fluid types, and both kinetic models were fitted to the data. Sub-analysis of HS infusion was also performed using a three-volume fluid space kinetic model taking into account the effects of osmotic fluid (27). The various kinetic models were compared, and the best model was selected using the log-likelihood test. The differential equation used for the 1-VOFS kinetic model was:

(2) $$\begin{array}{l} \frac{dv}{dt}=R_{0}-k_{10}\left(v-V\right) \end{array}$$

The differential equations used for the 2-VOFS kinetic model were:

(3) $$\begin{array}{l} \frac{dv_{c}}{dt}=\;R_{0}-k_{10}\left(v_{c}-V_{c}\right)-\;k_{12}\left(v_{c}-V_{c}\right)+k_{21}\left(v_{p}-V_{p}\right) \end{array}$$

(4) $$\begin{array}{l} \frac{dv_{p}}{dt}=\;k_{12}\left(v_{c}-V_{c}\right)-k_{21}\left(v_{p}-V_{p}\right) \end{array}$$

Serial plasma dilution and estimated urine output were used as input variables for VK analysis. Estimated urine output was included to stabilize the kinetic models. Intravenous infusion rates (*R*_0_) for PLA, HS, and HES were obtained directly from the prescribed fluid dosages. The average estimated urine outputs measured throughout the study were used to estimate *k*_10_ as follows:

(5) $$\begin{array}{l} \text{Elimination rate constant},\;k_{10}=\frac{\text{Urine output}}{\text{AUC for volume expansion}} \end{array}$$

The fixed parameters in the VK models, i.e., *V*_**c**_ for the 2-VOFS kinetic model, *V* for the 1-VOFS kinetic model, as well as the kinetic constants governing fluid distribution (*k*_12_ and *k*_21_) and elimination (*k*_10_), were generated using a specialized PK modeling and simulation software^21^ for non-linear mixed effects.

### Volume Kinetic and Covariate Analyses (Population Kinetics)

Fluid type, SAP, DAP, MAP, HR, age, and body weight were evaluated as potential covariates in the VK model. The continuous variables SAP, DAP, MAP, and HR were evaluated at every time point, while age and body weight were examined once for each individual data set. The first-order conditional estimation with extended least squares (FOCE-ELS) search routine was applied to provide maximum likelihood estimates for fixed and random effects. A search for trends in plots of random effects was also used to identify potentially significant covariates. A covariate was accepted if it significantly improved the curve-fit through reduction of -2 log likelihood (-2 LL) greater than the critical value of 6.64 (*p* < 0.01), the 95% confidence interval (CI) was statistically significant (excludes 1), and the inter-individual variability or coefficient of variation (CV) was <50%.

The influence of statistically significant covariates on VK was simulated using a computer software^20^. The best estimates of the model parameters were then inserted into Equations 2, 3 and 4. The analytical solutions to the differential equations of respective kinetic models were solved using the ordinary differential equations (optODE) function. The goodness-of-fit of the model was illustrated by comparing the observed experimental data with the model predicted data, with and without consideration of covariate effects.

### Plasma Volume Expansion, Half-Life, Fluid Potency, and Ideal Fluid Prescriptions

The magnitudes of the plasma volume expansion for PLA, HS, and HES were obtained from the respective plasma dilution-time curves and are presented as percentages. The half-life is represented by *t*_1**/**2_ in minutes and was obtained by the equation:

(6) $$\begin{array}{l} \text{Elimination half-life},t_{1/2}=\frac{\text{ln }2}{k_{10}} \end{array}$$

Fluid potency is an expression of fluid activity defined by the volume required to produce an effect of a given intensity. The comparative potencies of PLA, HS, and HES were illustrated using computer simulation of a standardized arbitrary fluid dose using VK parameter estimates generated from the statistically justified models. The ideal PLA, HS, and HES fluid prescriptions to achieve and maintain 15 or 30% plasma volume expansion were also simulated using VK parameter estimates generated from the respective statistically justified models.

## Statistics

Basic descriptive analysis was performed using an open source online statistical program^22^. The baseline Hb concentration, RBC count, and HCT were normally distributed and reported as mean ± standard deviation. The SAP, DAP, MAP, HR, and estimated total urine output were not normally distributed according to the Shapiro-Wilk test; logarithmic (base 10) transformation improved the data distribution of the estimated total urine output. Therefore, SAP, DAP, MAP, and HR are reported as median and interquartile range (IQR). Estimated total urine output and body weight are reported as mean ± standard deviation, while age is reported as median and range.

Volume kinetic and covariate analyses were performed by an independent individual (RH) using the specialized PK modeling and simulation software^21^ for non-linear mixed effects. Data sets from study subjects were analyzed collectively using a population analysis technique. The VK parameter estimates are reported as mean and 95% confidence interval (CI). Statistical significance was set at a *p* < 0.01. Model comparisons were performed, and statistically justified kinetic models were selected based on the reduction of -2 LL > 6.64 (*p* < 0.01). Forward and backward stepwise covariate search was used to identify statistically significant covariates through the reduction of -2 LL > 6.64 (*p* < 0.01).

## Results

Ten cats with a median age of 33 weeks (range 27–33) and mean body weight of 4.79 ± 0.69 kg completed all phases of the study with no adverse effects. All cats maintained normal activity levels and good appetite. No vomiting, diarrhea, abnormal respiratory patterns, or other concerning constitutional signs were recorded. Central venous catheters were replaced in four cats over a duration of 3 weeks due to inadvertent catheter dislodgment (*n* = 1), catheter site infection (*n* = 1), and non-salvageable catheter occlusion (*n* = 2). Throughout the study periods, all cats were normotensive with a median SAP of 116 mmHg (IQR 108–124) and median MAP of 86 mmHg (IQR 74–93; mean 84 ± 17 mmHg). The median HR was 157 beats per minute (IQR 139–187). The mean baseline Hb concentration and HCT at the start of the study were 99.5 ± 11.0 g/L and 30.3 ± 3.4%, respectively. The final Hb concentration and HCT recorded at the end of the study were 76.9 ± 9.5 g/L and 23.8 ± 2.8%, respectively.

Thirty experimental data sets (10 sets of PLA, 10 sets of HS, and 10 sets of HES) were acquired from 10 cats on 3 separate days. One set of PLA data was determined to be unsuitable for inclusion in the analysis based on serial plasma dilution that exceeded three standard deviations from the mean, likely due to spurious baseline measurement. Of the total 540 paired serial Hb concentrations and RBC counts, 37 (6.8%) measurements were removed due to incomplete data and 18 (3.3%) measurements from the unsuitable PLA data set were excluded from further analysis. The average CV for baseline Hb concentration and RBC count were 3.53 and 3.38%, respectively. The estimated total urine outputs for cats receiving PLA, HS, and HES were 37.3 ± 26.5, 16.2 ± 8.4, and 19.5 ± 14.8 mL, respectively. The mean infusion rates (*R*_0_) for PLA, HS, and HES were 6.5, 1.1, and 1.6 mL/min, respectively.

### Sample Collection

**Figure 2:**
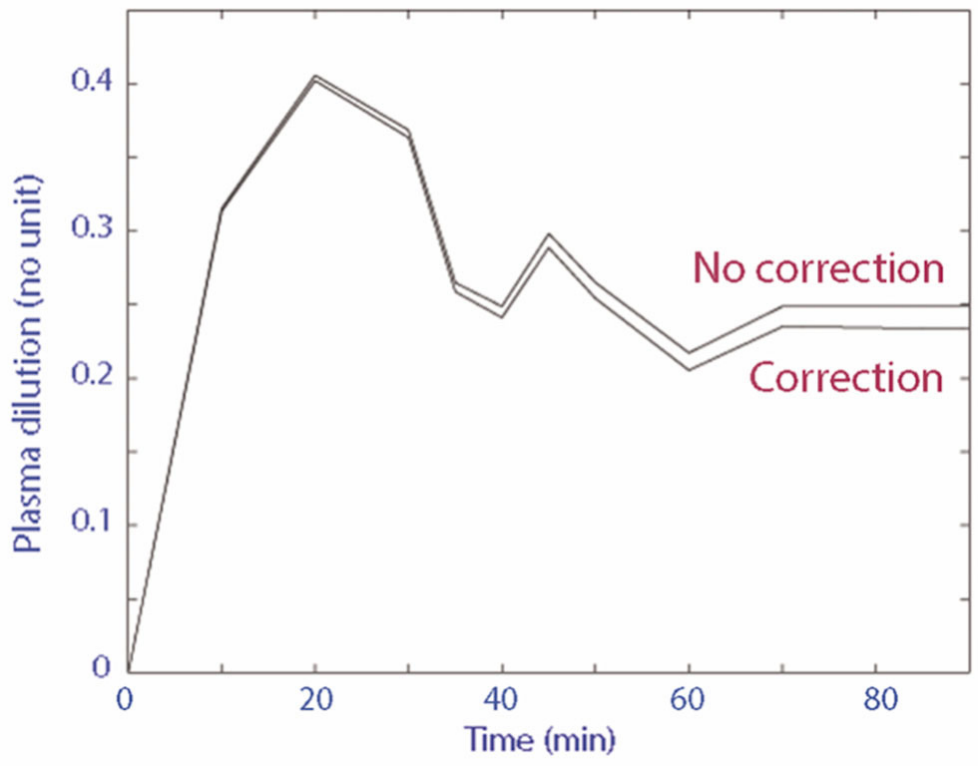
Plasma dilution-time curve with and without correction for Hb loss from serial blood sampling.

A total of 18 blood samples (~8 mL of whole blood) were collected from each cat for every individual experiment. The plasma dilution-time curve with and without correction for Hb loss from serial blood sampling of the three randomly selected experimental data sets diverged very little over time (Figure 2). In comparison to uncorrected plasma dilution, VK analysis using corrected plasma dilution (*n* = 3) resulted in lower *V*_*c*_ and higher *k*_10_ with the differences within an acceptable range of 2–10%.

### Group Fluid Analysis

**Figure 3:**
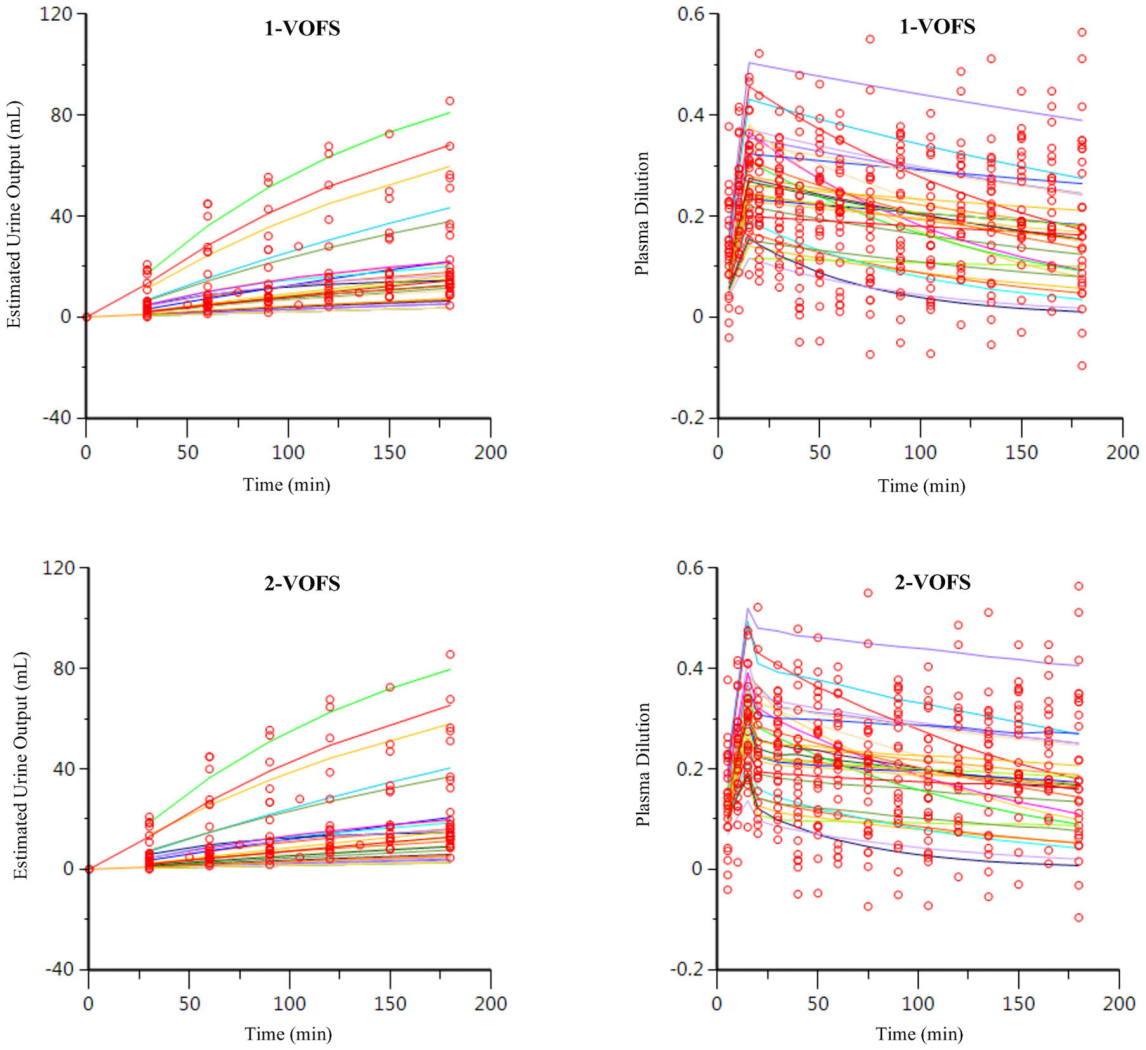
Goodness-of-fit plots of observation (open circles) and individual predictions (colored lines) against time for the 1-VOFS and 2-VOFS kinetic models of group fluid analysis.

Twenty-nine data sets, regardless of fluid type, were analyzed together, and both 1-VOFS and 2-VOFS kinetic models were fitted to the data. The VK parameter estimates of both kinetic models are summarized in Table 1. The goodness-of-fits of urine output and plasma dilution for both kinetic models are illustrated in Figure 3. The 2-VOFS kinetic model was statistically justified based on the reduction of -2 LL from 292 to 264 (*p* < 0.01), and thus is considered the better model for group fluid analysis.

**Table 1:** VK parameter estimates of both 1-VOFS and 2-VOFS kinetic models for group fluid analysis (*n* = 29) in healthy conscious cats.

**VK model**	**VK parameter**	**Estimate mean (95% CI)**	**CV**
1-VOFS	*V*, mL	409 mL (280–538)	16.05%
	*k*_10_, /min	0.003 /min (0.002–0.004)	17.59%
2-VOFS^*^	*V*_*c*_, mL	139 mL (83–195)	20.49%
	*k*_10_, /min	0.007 /min (0.004–0.010)	22.76%
	*k*_12_, /min	0.326 /min (0.117–0.534)	32.53%
	*k*_21_, /min	0.190 /min (0.128–0.252)	16.71%

* *denotes statistically justified model based on reduction of -2 LL > 6.64 (p < 0.01)*.

*VK, volume kinetic; CI, confidence interval; CV, coefficient of variation; 1-VOFS, one-volume fluid space; 2-VOFS, two-volume fluid space; V, volume of expandable body fluid space; k_10_, first-order elimination rate constant; V_c_, volume of expandable central fluid space; k_12_, central to peripheral intercompartmental rate constant; k_21_, peripheral to central intercompartmental rate constant*.

**Figure 4:**
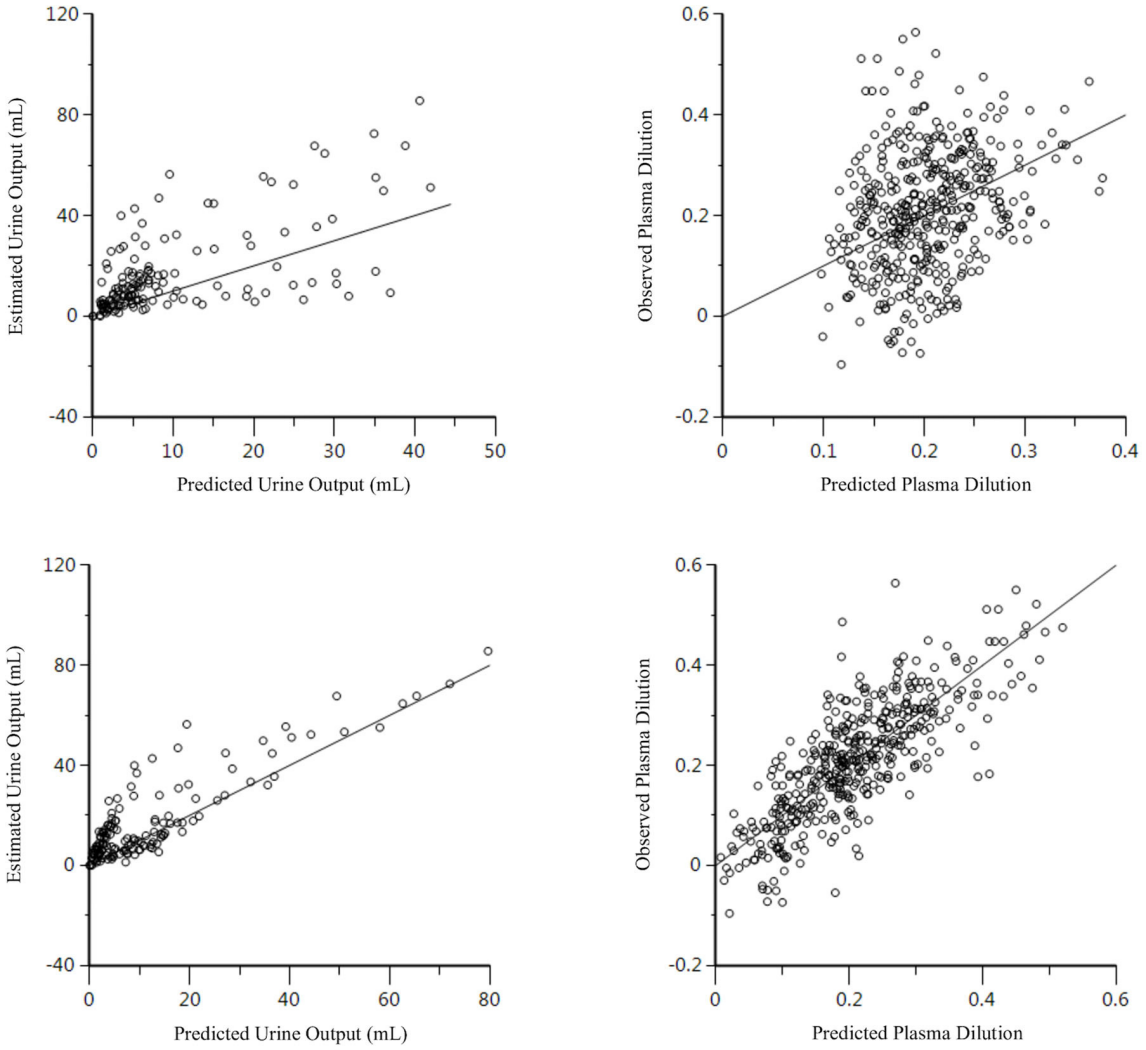
Residual plots for the 2-VOFS kinetic model of group fluid analysis before (top) and after (bottom) considering the covariate effects of fluid types and MAP.

In the 2-VOFS kinetic model, covariate analysis identified fluid type and MAP as significant covariates (*p* < 0.01) as supported by the improved residual plots (Figure 4). Subsequent sub-analysis demonstrated that fluid type was a covariate to *V*_*c*_, while MAP was a covariate to *k*_12_. With PLA as the reference fluid, the covariances between the different fluid types and *V*_*c*_ were −1.840 (95% CI −2.089 to −1.590) for HS and −1.298 (95% CI −1.621 to −0.975) for HES. Therefore, the *V*_*c*_ of individual fluid types can be expressed as the population *V*_*c*_ to the base of the natural logarithm of the covariance between a specific fluid type and *V*_*c*_:

$$\begin{array}{l} V_{c\text{ individual}}\\ =V_{c\text{ population}}\;e^{\text{covariance }\left(\text{e}.\text{g}.,\text{ PLA}:\;0,\text{ HS}:\;-1.840,\text{ HES}:\;-1.298\right)} \end{array}$$

The covariance between MAP and *k*_12_ was 0.053 (95% CI 0.029–0.077). The individual *k*_12_ can be expressed by the equation:

$$\begin{array}{l} k_{12\text{ individual}}=k_{12\text{ population}}{\left(\frac{MAP_{\text{ individual}}}{mean\;MAP}\right)}^{\text{covariance}} \end{array}$$

### Individual Fluid Analysis

**Figure 5:**
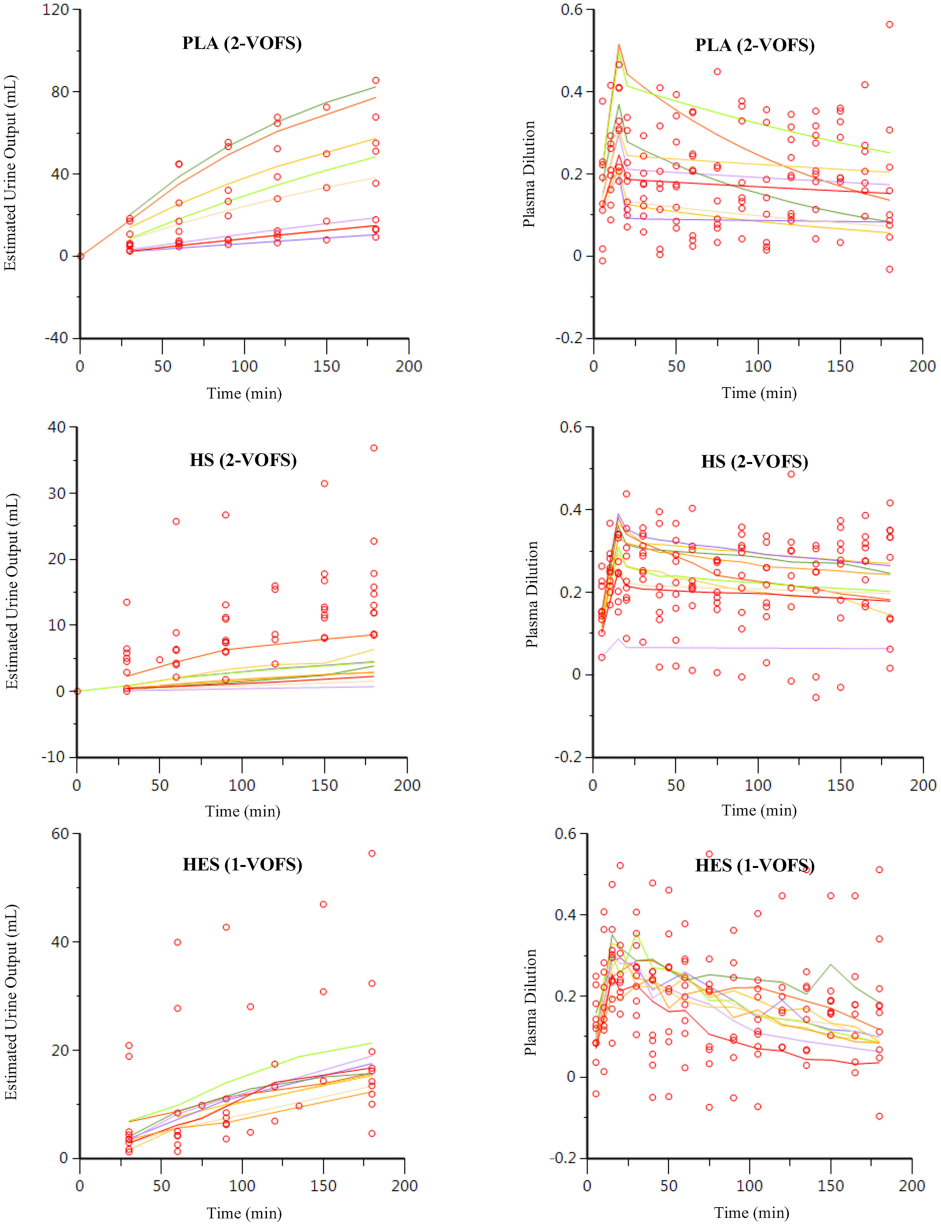
Goodness-of-fit plots of observation (open circles) and individual predictions (colored lines) against time for PLA, HS, and HES using respective statistically justified kinetic models.

The PLA data set (*n* = 9), HS data set (*n* = 10), and HES data set (*n* = 10) were analyzed separately according to fluid type, and both 1-VOFS and 2-VOFS kinetic models were fitted to the data. Sub-analysis of HS infusion was also performed using a three-volume fluid space kinetic model, taking into account the effects of osmotic fluid shift. The 2-VOFS kinetic model was statistically justified (*p* < 0.01) for PLA and HS, while the 1-VOFS kinetic model was statistically justified (*p* < 0.01) for HES based on the reduction of -2 LL > 6.64. The VK parameter estimates of statistically justified kinetic models for PLA, HS, and HES are summarized in Table 2. As depicted by Figure 5, the observed urine output values for HS and HES do not fit the values predicted by the statistically justified fitted models.

**Table 2:** VK parameter estimates of statistically justified VK models for individual fluid analysis in healthy conscious cats.

**IV fluid types**	**PLA mean** **(95% CI)**	**HS mean** **(95% CI)**	**HES mean** **(95% CI)**
Sample size, *n*	9	10	10
VK model^*^	2-VOFS	2-VOFS	1-VOFS
*V*_*c*_ or *V*	74.25 mL	32.41 mL	80.33 mL
	(28.93–119.57)	(22.11–42.72)	(58.59–102.07)
*k*_10_	0.014 /min	0.002 /min	0.007 /min
	(0.005–0.024)	(0.000–0.004)	(0.003–0.010)
*k*_12_	0.753 /min	0.141 /min	-
	(0.199–1.308)	(0.018–0.263)	
*k*_21_	0.161 /min	0.186 /min	-
	(0.089–0.233)	(0.048–0.323)	

* *denotes statistically justified model based on reduction of -2 LL > 6.64 (p < 0.01)*.

*IV, intravenous; PLA, balanced isotonic crystalloid; HS, 5% hypertonic saline; HES, 6% tetrastarch 130/0.4; CI, confidence interval; n, sample size; VK, volume kinetic; 2-VOFS, two-volume of fluid space; 1-VOFS, one-volume of fluid space; V_c_, volume of expandable central fluid space; V, volume of expandable body fluid space; k_10_, first-order elimination rate constant; k_12_, central to peripheral intercompartmental rate constant; k_21_, peripheral to central intercompartmental rate constant*.

Covariate analysis for PLA (*n* = 9) demonstrated trends in the plots of random effects, however, none of the tested covariates was statistically significant. The *t*_1/2_ for PLA was 49 min (95% CI 29–147). On the other hand, MAP was identified as a significant covariate (*p* < 0.01) to *k*_10_ for both HS and HES with a covariance of −2.865 (95% CI −5.350 to −0.380) and 2.904 (95% CI 0.017–5.790), respectively. The individual *k*_10_ can be expressed by the equation:

$$\begin{array}{l} k_{10\text{ individual}}=k_{10\text{ population}}{\left(\frac{MAP_{\text{ individual}}}{mean\;MAP}\right)}^{\text{covariance}} \end{array}$$

In consideration of MAP as a significant covariate to *k*_10_, the *t*_1/2_ for HS and HES when MAP was 84 mmHg were 319 min (95% CI 188–1054) and 104 min (95% CI 69–213), respectively.

### Plasma Volume Expansion and Fluid Potency

**Figure 6:**
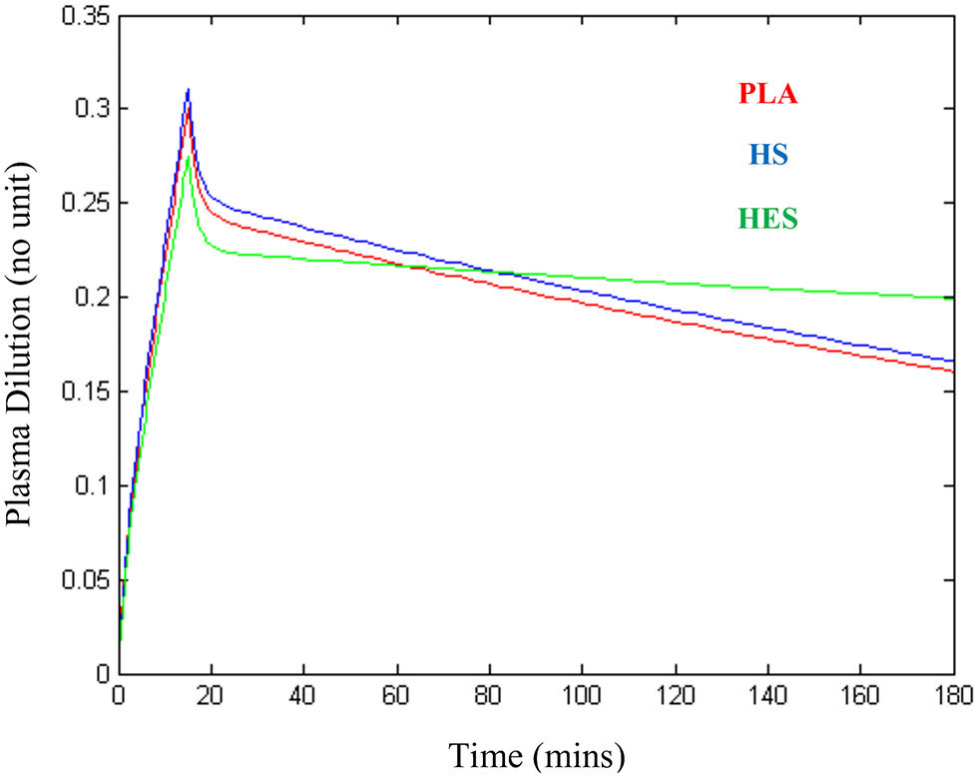
Plasma dilution-time curves illustrating approximately similar peak plasma expansion for 20 mL/kg of balanced isotonic crystalloid (PLA), 3.3 mL/kg of 5% hypertonic saline (HS), and 5 mL/kg of 6% tetrastarch 130/0.4 (HES) administered intravenously over 15 min in healthy conscious male cats.

**Figure 7:**
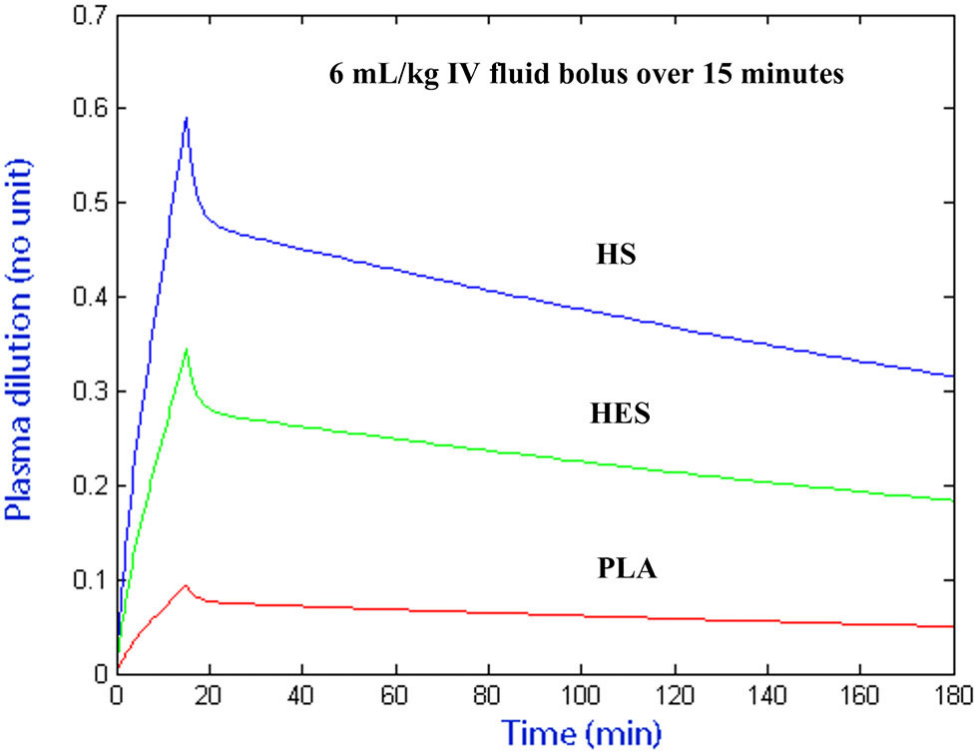
Computer simulated plasma dilution-time curves for PLA, HS, and HES that illustrates the different fluid potencies. Simulation performed using VK parameter estimates generated from the statistically justified 2-VOFS kinetic models for group fluid analysis.

The plasma dilution-time curves of PLA, HS, and HES administered at the presumed equipotent doses demonstrated an almost identical peak plasma volume expansion of ~27–30% (Figure 6). Computer simulation of a standardized 6 mL/kg bolus of each solution administered over 15 min using VK parameter estimates generated from the statistically justified 2-VOFS kinetic models for group fluid analysis illustrates the different fluid potencies in Figure 7. Of the IV fluids investigated, HS was six times more potent than PLA and 1.7 times more potent than HES. Meanwhile, HES was 3.5 times more potent than PLA.

### Simulation of Ideal Intravenous Fluid Infusions

**Figure 8:**
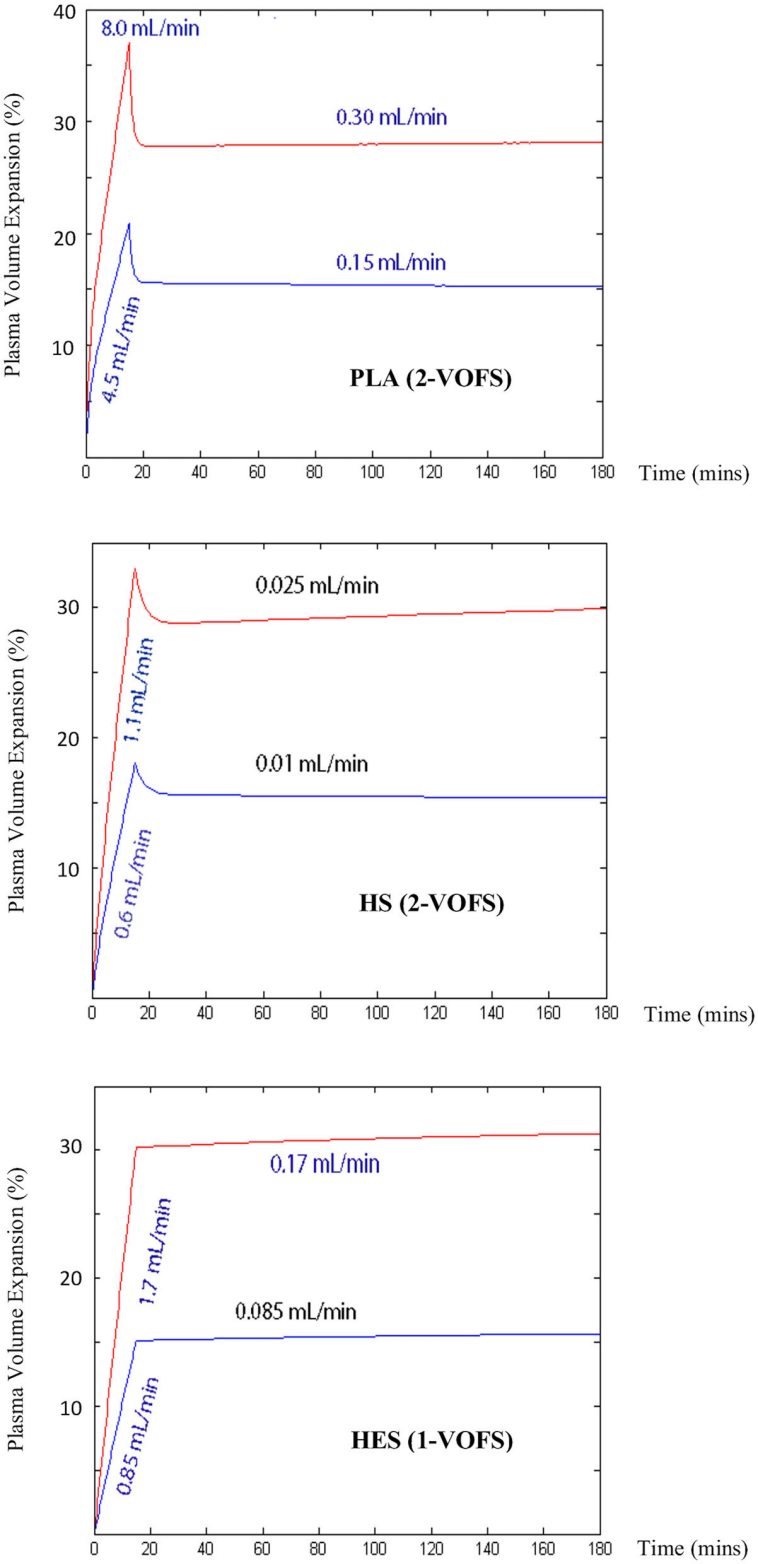
Computer simulated IV infusion rates for PLA, HS, and HES to achieve and maintain 15 or 30% plasma volume expansion in a 5 kg healthy conscious cat. Simulation performed using VK parameter estimates generated from respective statistically justified kinetic models.

The ideal PLA, HS, and HES fluid prescriptions to achieve and maintain 15 or 30% plasma volume expansion in a 5 kg healthy conscious cat simulated using VK parameter estimates generated from the respective statistically justified models are summarized in Table 3 and Figure 8.

**Table 3:** Simulated^*^ IV infusion rates of PLA, HS, and HES to achieve and maintain 15 or 30% plasma volume expansion in a 5 kg healthy conscious cat.

**IV fluid types**	**Desired plasma volume expansion**	**To achieve**		**To maintain**	
		***via*** **15-min bolus**		***via*** **CRI**	
		**(mL/kg)**	**(mL/min)**	**(mL/min)**	**(mL/hr)**
PLA	15%	12.0	4.0	0.15	9.0
	30%	24.0	8.0	0.30	18.0
HS	15%	1.8	0.6	0.02	1.2
	30%	3.6	1.2	0.04	2.4
HES	15%	3.0	1.0	0.04	2.4
	30%	6.0	2.0	0.08	4.8

*Simulated IV infusion rates, such as these, are important and may be clinically useful for practitioners*.

* *simulated using VK parameter estimates from 2-VOFS kinetic model of group fluid analysis*.

*IV, intravenous; CRI, constant rate infusion; PLA, balanced isotonic crystalloid; HS, 5% hypertonic saline; HES, 6% tetrastarch 130/0.4*.

## Discussion

This exploratory study demonstrated the feasibility and potential of VK modeling and analysis in evaluating the distribution, elimination, plasma volume expansion, half-life, potency, and ideal fluid prescriptions of PLA, HS, and HES in healthy conscious cats using serial Hb- and RBC- derived plasma dilution supported by estimated urine output. All cats successfully completed each phase of the experiment with only minor indwelling central venous catheter complications and tolerated the frequent blood sampling well. The frequency of measurement time points was sufficient to complete the VK analysis, although the 3-h observation period did not capture the entire return of plasma dilution to baseline. Kinetic models were successfully fitted to all experimental data sets and a novel approach of VK analysis, where all fluids were analyzed in a unified model, produced similar findings as the conventional individual fluid analysis approach. Overall, the results generated from this study were logical and physiologically sound. The demonstration of similar peak plasma volume expansion following administration of approximately equipotent fluid doses, as well as half-life and fluid potency that conform to expectations suggest that VK modeling and analysis has the potential to investigate IV fluid kinetics and body water physiology in cats.

Population kinetics is a PK analysis technique that identifies and quantifies sources of variability in substance concentrations between individuals (e.g. age, body weight, physiologic variables, or disease states), which is useful for therapy customization (28). In this type of analysis, all data from all individuals is considered at the same time in a unified model using a non-linear mixed effects modeling approach (29). Although analyzing all fluid types together seems counterintuitive, covariate analysis was able to identify fluid type and MAP as key covariates and determine the degree of variability on the kinetic model. Insertion of relevant covariances into the generated formulas allowed simulation of individual fluids, while a unified model enabled a simplified view of the overall experimental outcome. Analyzing different types of IV fluids together in a unified model has not been previously reported; however, the 2-VOFS kinetic model was successfully fitted to the experimental data and was statistically justified. Regardless of whether group or individual fluid analyses were performed, the overall findings and conclusions were similar, suggesting that multiple approaches to VK modeling and analysis can be considered along with increasing confidence in the models due to their robustness.

When individual fluid analyses were performed, the 2-VOFS kinetic model was better fitted to PLA, while the 1-VOFS kinetic model was appropriate for HES in our population of healthy conscious cats. This is consistent with VK studies of healthy conscious human volunteers (16, 27, 30), except in situations where rapid fluid elimination allowed the 1-VOFS kinetic model to sufficiently describe the kinetics of crystalloid infusions (16, 27, 31). The three-volume fluid space kinetic model was explored for HS however it failed to add notable advantages without compromising model simplicity. The 2-VOFS model was found to be appropriate for HS in our study, similar to the findings in human volunteers (27).

When compared to the individual fluid analysis, the goodness-of-fit plots generated from group fluid analysis revealed better agreement between the observed experimental data and model predicted data, suggesting that group fluid analysis may have more precision. The poor agreement between the observed and model predicted urine output of individual fluid analyses, in particular for HS and HES (Figure 5), may be a result of inaccuracies in estimating urine output using point-of-care ultrasonography. However, it is interesting that the imprecisions only involved HS and HES infusions, possibly due to the small volumes of urine generated in these experiments. A second elimination compartment was tested *post-hoc* without improvement of these models, suggesting that the estimated urine output truly represents fluid elimination. Regardless, urine output is not a crucial input variable in VK analysis although it serves to stabilize the VK model, if available (32). Alternative explanations to the discrepancy between estimated urine output and model predicted urine output for HS and HES include increased glomerular filtration rate as a result of fluid shifts from other parts of the body (33), effects of natriuresis (27, 34), or decreased water reabsorption from the presence of filtered oncotic macromolecules (30). The effects of natriuresis are considered less likely given the equimolar sodium ion administered for HS and PLA.

In comparison to healthy humans, the rates of bi-directional fluid distribution from the central to the peripheral compartment (*k*_12_) and vice versa (*k*_21_), were found to be very fast in our study subjects. The distribution phase of PLA and HS in our healthy cats were between 5 and 10 min (Figure 6), at least 3–4 times faster than the reported 25–30 min in healthy human adults receiving crystalloid infusions (18, 35) and healthy anesthetized dogs (36). These findings are based on the plasma dilution-time curves, whereby the distribution phase is represented by the initial steep decline in plasma dilution at the end of the fluid boluses. In humans, the lag time for IV fluids to equilibrate between the plasma and the interstitium allows for cumulative plasma volume expansion up to 50–70% (37, 38) by the end of a fluid bolus. It is possible that fluid distribution occurred while IV infusion was still ongoing in our cats. When IV fluids were analyzed individually in our study, the distribution of PLA from the central to the peripheral compartment was five times faster than HS. We believe this may, in part, be due to the increase in transcapillary hydrostatic pressure and fluid filtration related to the total volume of administered fluid. This rapid distribution phase in healthy cats suggests that cats have a more pliable and compliant interstitial matrix (39) than other species.

Interestingly, in our population of healthy conscious cats, the rate of fluid elimination as described by *k*_10_ was found to be very slow for all individual fluid types, in contrast to healthy conscious sheep (40, 41) and healthy human volunteers (16, 27, 31). A marked reduction in fluid elimination is reported in anesthetized human patients undergoing various surgical procedures (14, 18), and is also documented in experimental anesthetized sheep (40, 41). When renal excretion fails to increase in proportion to the volume of infused fluid, whether under general anesthesia (40–42) or when subjected to supraphysiologic infusions (43), low fluid elimination augments plasma volume expansion and increases the risk of interstitial edema formation from the surplus infused volume.

Furthermore, our data suggest that the rate of fluid elimination for PLA was twice as rapid as HES and up to seven times faster in comparison to HS. The *t*_1/2_ of PLA, HS, and HES in our population of healthy conscious male cats were found to be 49, 319, and 104 min, respectively. The *t*_1/2_ of PLA is comparable with studies in human volunteers, where the median *t*_1/2_ was reported to be between 20 and 60 min depending on gender, stress levels, and hydration status (16, 31, 37, 44–46). For colloids, the oncotic macromolecule *t*_1/2_ of 130/0.4 HES in healthy human volunteers was discovered to be much prolonged at 12 h (47) and 16 h (48) in those with renal impairment. Of note, these *t*_1/2_ relate to the HES macromolecules and not the duration of plasma volume expansion, which the latter is of key interest to clinicians. In VK studies performed in healthy human volunteers, the *t*_1/2_ of 130/0.4 HES which reflected the actual decay of plasma volume expansion was 2 h (16, 30), which is similar to our reported *t*_1/2_ of 104 min. This is also consistent with the reported systemic circulation *t*_1/2_ in the product monograph (49). Direct comparison of the *t*_1/2_ of HS with other studies is not possible due to the different strength utilized in our study. Based on our traditional understanding of the physiological mechanism of HS in the body (33), we expect HS to have a shorter *t*_1/2_ and more rapid elimination due to the effects of natriuresis (34). However, given that we administered equimolar sodium load for both HS and PLA, the prolonged *t*_1/2_ of 319 min and slower elimination rate might be as a result of renin-aldosterone-angiotensin-system activation from detection of increased chloride ions.

Although cats remained normotensive throughout the experiments, MAP was found to be a significant covariate for fluid distribution out of the central compartment (*k*_12_) in the group fluid analysis, and fluid elimination (*k*_10_) for HS and HES. As MAP increases, transcapillary fluid filtration contributes to the increased fluid distribution while stimulation of baroreceptors and activation of renal blood pressure regulation feedback system promotes fluid elimination. The only unexpected finding was a negative covariance between MAP and *k*_10_ for HS, in which an increase in MAP decreases fluid elimination. The reason for this phenomenon is uncertain at this time.

There are many ongoing debates about the volume efficacy and plasma volume expansion effects of various IV fluids. Data pertaining to the comparative intravascular volume expansion effectiveness of different fluid types in humans and dogs have been thoroughly reviewed (50, 51). Unfortunately, limited scientific data are available in cats, hence we prescribed fluid doses that we considered represented approximately equipotent volume expansion effects. Interestingly, the plasma-dilution time curves of 20 mL/kg PLA, 3.3 mL/kg HS, and 5 mL/kg HES administered over 15 min generated an almost identical peak plasma volume expansion of ~27–30%. The 4:1 ratio of isotonic crystalloid to colloid appeared to be valid in our population of healthy cats. This is different from the results of recent critically ill human clinical trials (52–55) that showed a cumulative total crystalloid to colloid infusion volume ~1.2–1.4:1 ratio. When microvascular circulation is considered, our population of healthy cats are thought to have an uncompromised endothelial glycocalyx layer which may have preserved the volume expanding effects of HES (56–58). Conversely, the argument that our large-volume and rapid PLA infusion rate in euvolemic cats may have resulted in direct endothelial glycocalyx damage leading to instantaneous fluid distribution is also possible but considered unlikely since all IV fluid types assessed distributed swiftly in general. Rapid large-volume crystalloid resuscitation has been associated with increased biomarker glycocalyx shedding in a recent canine hemorrhagic shock model (59). Hypervolemia has also been suggested to promote atrial natriuretic peptide release that contributes to endothelial glycocalyx shedding (60), although the subject matter is controversial (61). Plasma atrial natriuretic peptide levels were not measured or investigated in our study.

Following computer simulation of a standardized arbitrary IV fluid dose for all 3 fluids, HS was found to be six times more potent than PLA and 1.7 times more potent than HES, while HES was 3.5 times more potent than PLA. In general, the potencies of these fluids were in line with findings from other human and canine studies (18, 27, 36), albeit experimental differences exist in the use of different strengths of hypertonic saline and types of synthetic colloid solutions. Unlike the previous canine study (36), our study had the advantage of equipotent fluid doses and plasma volume expansion effect, thus removing the confounding effect of total fluid volume administered, allowing direct comparison between individual fluids.

In summary, the data generated from VK analysis in this population of healthy conscious cats support anecdotal reports (10) that cats are more susceptible to the harmful effects of fluid therapy. Our work using this novel approach to IV fluid analysis suggests that cats are different from other species studied, in that commonly utilized IV fluids which we investigated as part of this study protocol distribute more rapidly into the peripheral compartments yet are eliminated more slowly from the body. Low fluid elimination augments plasma volume expansion and increases the risk of interstitial edema formation from the surplus infused volume. This important finding may be the key to the increased susceptibility of this species to fluid overload. Computer simulation using VK parameter estimates generated from pooled and individual fluid analyses highlighted the need for substantial reduction in the constant rate infusion following the initial fluid bolus of all three fluids in order to prevent fluid overload and to maintain the desired plasma volume expansion at steady state. This specific recommendation, however, must be taken in context and is only applicable to healthy conscious euvolemic cats without any ongoing fluid losses. Additional work is warranted to determine the IV fluid kinetics in dehydrated, hypovolemic, or hypotensive cats that represent the clinical population that would be subjected to IV fluid resuscitation followed by ongoing maintenance fluid therapy.

### Study Limitations and Future Recommendations

As a preliminary study, our study had a number of limitations. Firstly, the Hb-dilution technique used in this study to quantify plasma dilution has not been validated in cats. This technique, however, has correlated well with other plasma volume determination techniques that utilized radio-iodinated serum albumin (62), blood water desiccation (16), Evans Blue dye (40), and indocyanine green dye (41) in humans and sheep. Secondly, our study subjects were not splenectomised therefore the effect of RBC sequestration and release on plasma dilution determination is unknown. Feline splenic contraction is reported to be brief and transient, lasting only 2 min and reaches a steady-state PCV within 20 min (63). Regardless, initiatives to reduce stress and sympathetic stimulation were implemented throughout each experiment. Recently, the use of trazodone in healthy cats was discovered to significantly decrease systolic blood pressures without resulting in hypotension by reduction of systemic vascular resistance *via* α_1_-adrenergic antagonism (64), contrary to previous report in the literature (65) that was available at the time of our study inception. Therefore, it is possible that trazadone may have had some effects on MAP or pre-capillary sphincter tone, and indirectly influenced our study results. Until additional evidence is available, gabapentin may be considered as an alternative oral sedative agent (66) to trazodone to avoid potential influence on fluid kinetics.

As with most veterinary studies, our sample size was small (*n* = 10) but considered acceptable for a preliminary study. For this type of experiment, erroneous baseline measurements had considerable consequences as evidenced by the exclusion of an entire data set even though subsequent serial measurements were appropriate, which further reduced our study sample size. In addition, the average CV of baseline Hb concentration of 3.53% and RBC of 3.38% obtained in our study were higher than the recommended 1% for VK studies (18). Accurate measurements and high-precision laboratory analysis are important to reduce between-sample variability that would affect serial plasma dilution determination and subsequent VK analysis (18). Homogeneity within the sampled blood achieved by adequate mixing is crucial to the quality of analytical results. Specimens idled for over 30 min have a tendency to settle despite anticoagulation and if not thoroughly mixed, Hb values can be overestimated or underestimated depending on whether the concentrated cellular layer or plasma-diluted layer was measured (67). Since simultaneously measured RBCs are diluted similarly as Hb during IV fluid loading but quantified using a different technique by the automated hematology analyzer, the mean value for Hb-derived plasma dilution and RBC-derived plasma dilution was used as the overall plasma dilution to improve precision and accuracy (26). This is important as measurement error was shown to have compounding and exponential effects on serial plasma dilution calculation (68).

For the same reasons above, corrections for Hb loss from blood sampling or hemorrhage have been recommended prior to the curve-fitting procedure as serial blood sampling can result in sufficient Hb loss creating a “false dilution” that is unrelated to IV fluid therapy (18). This, however, was not performed in our study since the blood sample volume at each sampling time point was small (0.4 mL). In addition, an assumption of initial blood volume is necessary to account for sampling losses (17), which meant having to introduce more uncertainties and potential source of error into the model. Although the degree of error associated with the use of varying initial blood volume is acceptable in humans (26, 37), the feline blood volume reported in the current veterinary literature is based on a small number of past experiments (63, 69, 70) and ranges widely from 40 to 66 mL/kg (71, 72) depending on whether cats are splenectomised or not (63, 70). The plasma dilution-time curve of 3 randomly selected experimental data sets did not differ substantively following correction of Hb loss. However, the plasma dilution-time curves continued to diverge gradually over time (Figure 2), likely due to compounding effects as more Hb was lost from repetitive blood sampling.

Frequent blood sampling over the duration of 3 weeks led to documented iatrogenic anemia as low as HCT of 20% by the end of the study. The implication of repeated blood sampling was small in our study as each study subject served as their own matched control in a cross-over design and new baseline measurements were established for each individual experiment. Although cats in this cross-over experimental study tolerated the frequent blood samplings well, if unlimited resources are available, a longer between-experiment period should be considered to allow recovery from iatrogenic anemia. However, unless a non-invasive method of serial Hb measurement is developed and validated for VK studies, VK modeling and analysis would likely remain a research tool as the requirement for repetitive invasive Hb sampling precludes it from widespread clinical use. For future studies, we recommend the exploration of non-invasive Hb monitoring technology for potential translation of VK modeling and analysis to the clinical population. Exploration of bioelectrical impedance technology as an adjunct tool to further support and complement the findings of VK analysis is also suggested.

Given that our study subjects came from a homogenous population of healthy male intact young cats, the results of this study cannot be translated to clinical patients. Lastly, VK modeling and analysis can be an inherently complex and sophisticated research method to characterize the PK of IV fluids. To use the information generated effectively, one must possess some mathematical and statistical background, combined with an understanding of biology, pharmacology, and physiology. Data collection is simple and straightforward; however, data analysis and interpretation involve a steep learning curve for those who are unfamiliar with these concepts. However, some coaching and training in these areas would accelerate this learning curve.

## Conclusion

In conclusion, our study demonstrated that VK modeling and analysis is a feasible research tool that has the potential to provide valuable data on IV fluid distribution, elimination, half-life, plasma volume expansion, fluid efficacy, and body water physiology in cats. The preliminary results generated from VK analysis revealed rapid distribution but slow elimination of IV fluids in healthy conscious cats that is in agreement with anecdotal reports related to susceptibility of cats to fluid overload and warrants further investigation. The prescribed fluid doses of 20 mL/kg PLA, 3.3 mL/kg HS, and 5 mL/kg HES administered over 15 min produced approximately similar peak plasma volume expansion of 27–30%. The half-life and potency of HS was the longest and greatest followed by HES, while PLA had the shortest half-life and was least potent. Simulation of ideal fluid prescriptions to achieve and maintain a 15 or 30% plasma volume expansion at steady state in a 5 kg healthy conscious euvolemic cat revealed the importance of substantial reduction in constant rate infusion following initial IV fluid bolus in this population.

## Data Availability Statement

The raw data supporting the conclusions of this article will be made available by the authors, without undue reservation.

## Ethics Statement

The animal study was reviewed and approved by the University of Guelph's Animal Care Committee (Animal Utilization Protocol 3668) and adhered to the University of Guelph Animal Care Policy and Procedures, the provincial legislation and regulations of the Animals for Research Act, and the national guidelines and policies of the Canadian Council on Animal Care. All individuals with direct animal involvement in this study completed the University of Guelph's Animal User Training Program.

## Author Contributions

XY, SB, and AB contributed to the conception and design of the study. XY and SB conducted the research. XY acquired and organized the data and wrote the first draft of the manuscript. RH performed the data analysis. XY, SB, and RH contributed to data interpretation. SB, AB, and RH provided critical revision of the manuscript. All authors read and approved the final manuscript.

## Conflict of Interest

The authors declare that the research was conducted in the absence of any commercial or financial relationships that could be construed as a potential conflict of interest. The handling editor WM declared a past supervisory role with one of the authors.

## Footnotes

^1^ThunderShirt®, ThunderWorks, Durham, NC, USA.

^2^MILACATH® guidewire, single lumen, 20 Ga × 12 cm with integrated extension set, MILA International, Inc., Florence, KY, USA.

^3^Torbugesic®, Zoetis Canada Inc., Kirkland, QC, Canada.

^4^Dexdomitor®, Zoetis Canada Inc., Kirkland, QC, Canada.

^5^Propofol Injection, Fresenius Kabi Canada Ltd., Toronto, ON, Canada.

^6^AErrane Isoflurane USP, Baxter Healthcare Corporation, Deerfield, IL, USA.

^7^Heparin Sodium Injection USP, Fresenius Kabi Canada Ltd., Toronto, ON, Canada.

^8^Antisedan®, Zoetis Canada Inc., Kirkland, QC, Canada.

^9^Sterilin, Inopro Inc., Repentigny, QC, Canada.

^10^Activase® rt-PA, Hoffmann-La Roche Limited, Mississauga, ON, Canada.

^11^TEVA Trazodone, TEVA Canada Limited, Toronto, ON, Canada.

^12^Feliway®, CEVA Animal Health Inc., Cambridge, ON, Canada.

^13^PLASMA-LYTE A Injection, Baxter Corporation, Mississauga, ON, Canada.

^14^5% Sodium Chloride Injection USP, Baxter Corporation, Mississauga, ON, Canada.

^15^VOLUVEN®, Fresenius Kabi Canada Ltd., Richmond Hill, ON, Canada.

^16^VET-PRO VIP 2000^TM^ Veterinary Infusion Pump, Caesarea Medical Electronics Ltd., Staufenburgstr, Lichtenstein, Germany.

^17^Urbaniak, G.C., and Plous, S. (2013). Research Randomizer (Version 4.0) [Computer software]. Retrieved on June 24, 2017, Available online at: http://www.randomizer.org/.

^18^Siemens ADVIA 2120i Hematology Analyzer, Siemens Healthcare GmbH, Henkestr, Erlangen, Germany.

^19^Cardell® Veterinary Vital Signs Monitor, Model 9401, Midmark, Tampa, FL, USA.

^20^MATLAB®, R2013b, MathWorks® Inc, Natick, MA, USA.

^21^Phoenix® NLME™, version 1.3, Certara, St. Louis, MO, USA.

^22^RStudio Team (2018). RStudio: Integrated Development for R. (Version 1.2.1206.2) [Computer software]. Retrieved on April 14, 2019, from http://www.rstudio.com/.

## Abbreviations

(0): at baseline
(*t*): at time *t*
1-VOFS: one-volume fluid space
2-VOFS: two-volume fluid space
CI: confidence interval
CV: coefficient of variation
DAP: diastolic arterial pressure
Hb: hemoglobin
HCT: hematocrit
HR: heart rate
HES: 6% tetrastarch 130/0.4
HS: 5% hypertonic saline
IQR: interquartile range
IV: intravenous
*k*_10_: first-order elimination rate constant
*k*_12_: central to peripheral intercompartmental rate constant
*k*_21_: peripheral to central intercompartmental rate constant
LL: log likelihood
MAP: mean arterial pressure
PK: pharmacokinetics
PLA: balanced isotonic crystalloid
RBC: red blood cell(s)
SAP: systolic arterial pressure
*t*_1/2_: elimination half-life
*V*: volume of expandable body fluid space
*v*: volume of expanded body fluid space
*v*−*V*: volume expansion
*c*: central
*p*: peripheral
VK: volume kinetics.
